# Supplementary material for: Multi-Generation Ecosystem Selection of Rhizosphere Microbial Communities Associated with Plant Genotype and Biomass in Arabidopsis thaliana
Source: Microorganisms. 2023 Dec 6;11(12):2932. doi: 10.3390/microorganisms11122932 (PMC10745315; doi:10.3390/microorganisms11122932)
Supplement: Supplementary file 1 [file microorganisms-11-02932-s001.zip › microorganisms-2725644-supplementary.pdf]

SUPPLEMENTARY FIGURES AND TABLES

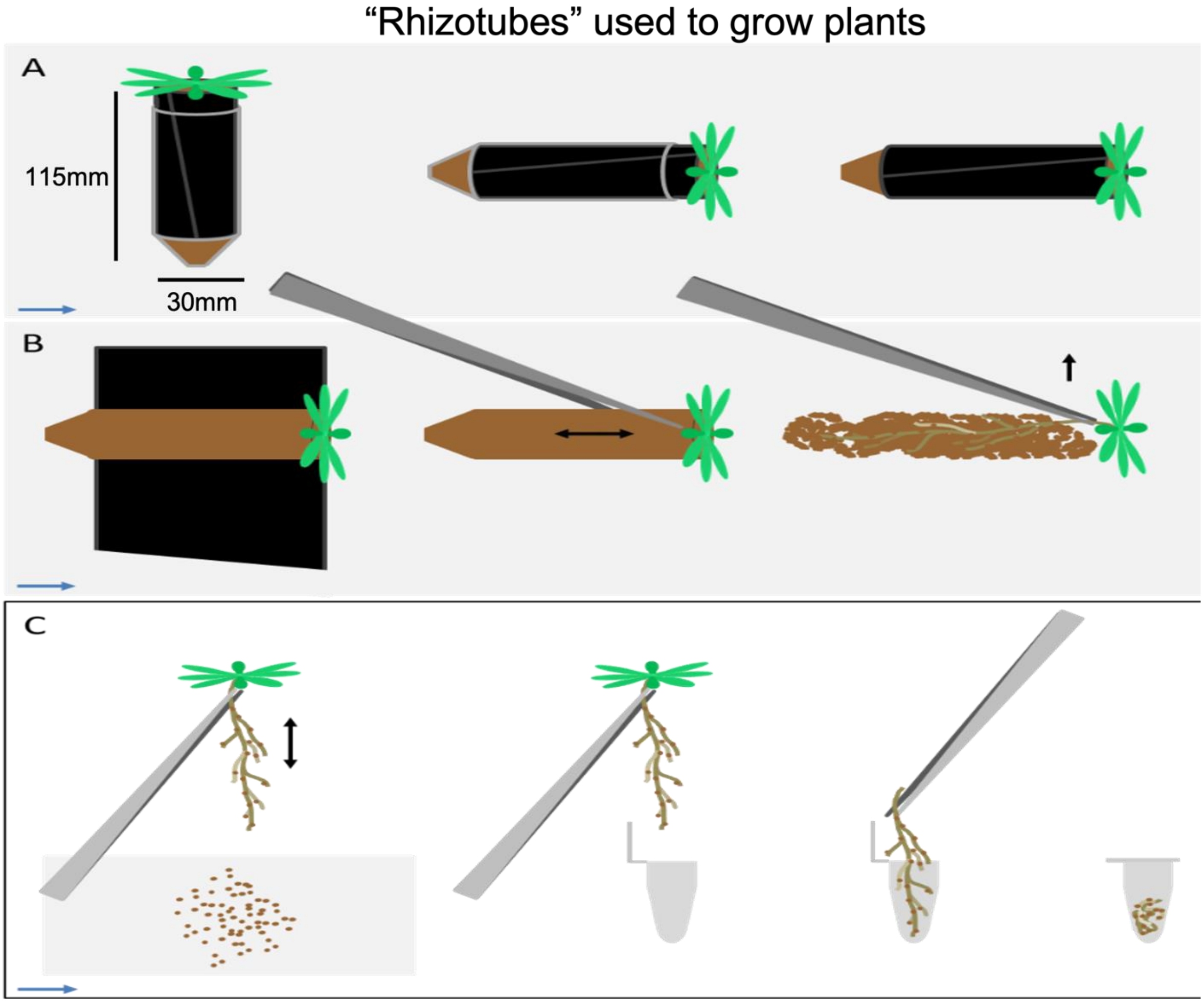

Supplementary Figure S1. Rhizotube shchematic. The purpose of this illustration is to demonstrate how the custom-made rhizotubes facilitate the collection of plant rhizosphere. The black insert within the tube is unwrapped to extract the rhizosphere, which is then separated from the bulk soil by shaking the roots. After removing the above-ground portion of the plant, the root-soil complex (comprising the rhizosphere and the endosphere) was obtained by shaking off the excess soil and then placed in a sterile 5ml tube and bulk soil samples were moved to a sterile Ziplock bag. Both were immediately transferred to dry ice and then stored at -80oC for DNA analysis.

.

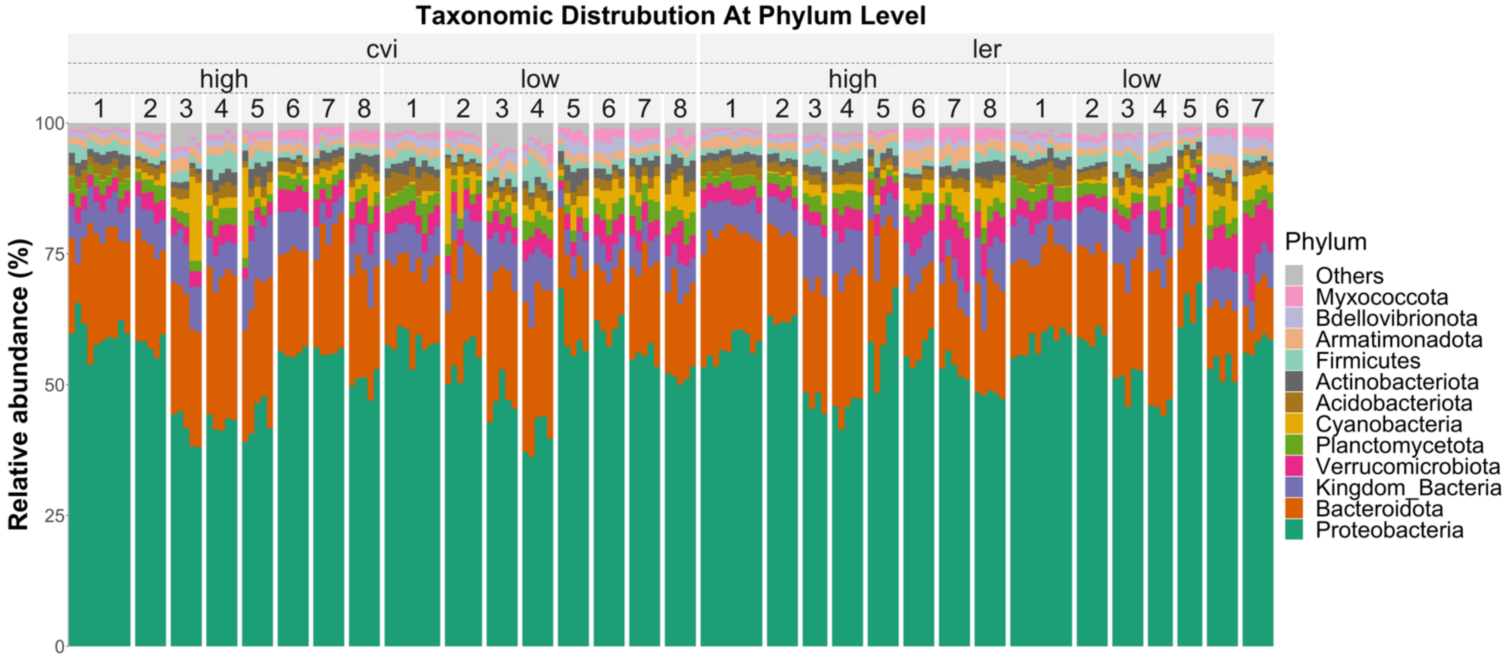

Supplementary Figure S2. Taxonomic distribution of the microbial community represented in terms of the relative abundance at the Phylum level. The plot shows eight generations (1-8) for the high and low biomass treatments of both the Cvi (left) and L*er* (right) genotypes of *Arabidopsis thaliana*. The microbial community is dominated by *Proteobacteria*, *Bacteroidetes*, *Verrucomicrobia, and Planctomycetes* which together comprise nearly 73% of the bacterial community.

Generation	group1	group2	p	p.signif
Gen4	High	Low	1.222020e-02	*
Gen5	High	Low	1.032381e-02	*
Gen7	High	Low	3.562072e-05	****

Supplementary Table S1 Statistical comparisons of biomass for L*er* samples (t.test p-value * < 0.05; **<0.01; *** <0.001).

Generation	group1	group2	p	p.signif
Gen4	High	Low	1.275260e-02	*
Gen7	High	Low	4.123576e-02	*
Gen8	High	Low	6.758467e-08	****

Supplementary Table S2 Statistical comparisons of biomass for Cvi samples (t.test p-value * < 0.05; **<0.01; *** <0.001).

	Df	Sum Sq	Mean Sq	F value	Pr(>F)	
Generation	7	158.13	22.590	333.054	<2e-16	***
Lineage	1	0.01	0.009	0.130	0.719	
Regime	1	0.01	0.011	0.169	0.681	
Residuals	160	10.85	0.068			

Signif. codes: 0 ‘***’ 0.001 ‘**’ 0.01 ‘*’ 0.05 ‘.’ 0.1 ‘ ’

Supplementary Table S3. ANOVA for Observed alpha diversity index shows a significant influence of generation on alpha diversity. Lineage is genotype and Regime is biomass treatment. Design (Observed ~ Generation + Lineage + Regime) Df: degrees of freedom; SumOfSqs : sum of squares; Mean sq: mean squares; F: F statistic; Pr(>F): p-value.

	Df	Sum Sq	Mean Sq	F value	Pr(>F)	
Generation	7	132.11	18.873	82.238	<2e-16	***
Lineage	1	0.01	0.009	0.040	0.841	
Regime	1	0.16	0.160	0.695	0.406	
Residuals	160	36.72	0.229			

Signif. codes: 0 ‘***’ 0.001 ‘**’ 0.01 ‘*’ 0.05 ‘.’ 0.1 ‘ ’

Supplementary Table S4. ANOVA for Shannon alpha diversity index shows a significant influence of generation on alpha diversity. Lineage is genotype and Regime is biomass treatment. Design (Observed ~ Generation + Lineage + Regime) Df: degrees of freedom; SumOfSqs : sum of squares; Mean sq: mean squares; F: F statistic; Pr(>F): p-value.

```
Type III Analysis of Variance Table with Satterthwaite's method
                          Sum Sq Mean Sq NumDF DenDF F value   Pr(>F)
Lineage                   0.0035  0.0035     1   139  0.0065 0.936050
Regime                    0.0945  0.0945     1   139  0.1770 0.674654
Generation                1.4097  0.2014     7   139  0.3771 0.914458
Lineage:Regime            4.6552  4.6552     1   139  8.7162 0.003704 **
Lineage:Generation       11.2206  1.6029     7   139  3.0013 0.005765 **
Regime:Generation         2.8505  0.4072     7   139  0.7624 0.619688
Lineage:Regime:Generation 9.7397  1.6233     6   139  3.0393 0.007990 **
---
Signif. codes:  0 ‘***’ 0.001 ‘**’ 0.01 ‘*’ 0.05 ‘.’ 0.1 ‘ ’ 1
```

Supplementary Table S5. Linear mixed effect model for Shannon diversity index. Shows a significant effect of the generation and interaction term with genotype (“Lineage”) and biomass treatment (“Regime”). Design lmer (DiveristyMetric, Genotype*Generation*Regime, (1|Generation)) Sum Sq: sum of squares; Mean sq : mean squares; NumDF: numerator degrees of freedom; DenDF: denominator degrees of freedom; F value: F statistic; Pr(>F): p-value.

```
adonis2(formula = wunifrac ~ Regime + Lineage, data = meta, permutations = 999
         Df  SumOfSqs      R2      F Pr(>F)
Regime    1 0.0020294 0.13730 6.3126  0.001 ***
Lineage   1 0.0008563 0.05794 2.6637  0.038 *
Residual 37 0.0118951 0.80476
Total    39 0.0147809 1.00000
---
Signif. codes:  0 ‘***’ 0.001 ‘**’ 0.01 ‘*’ 0.05 ‘.’ 0.1 ‘ ’ 1
```

Supplementary Table S6. PERMANOVA weighted UniFrac distances for generation 1. The R^2 values were computed using the PERMANOVA test with the adonis2 function for generation 1 for weighted UniFrac distances. Genotype is referred to as “Lineage” and biomass treatment as “Regime”. Model (distance.matrix~biomass treatment +genotype).

```
adonis2(formula = wunifrac ~ Regime + Lineage, data = meta, permutations = 999
         Df  SumOfSqs      R2      F Pr(>F)
Regime    1 0.0033556 0.22498 5.0755  0.006 **
Lineage   1 0.0036257 0.24309 5.4840  0.001 ***
Residual 12 0.0079337 0.53193
Total    14 0.0149149 1.00000
---
Signif. codes:  0 ‘***’ 0.001 ‘**’ 0.01 ‘*’ 0.05 ‘.’ 0.1 ‘ ’ 1
```

Supplementary Table S7. PERMANOVA weighted UniFrac distances for generation 8. The R^2 values were computed using the PERMANOVA test with the adonis2 function for generation 8 for weighted UniFrac distances. Genotype is referred to as “Lineage” and biomass treatment as “Regime”. Model (distance.matrix~biomass treatment +genotype).

```
adonis2(formula = unifrac ~ Regime + Lineage, data = meta, permutations = 999)
         Df SumOfSqs      R2      F Pr(>F)
Regime    1   0.2022 0.03895 1.5537  0.001 ***
Lineage   1   0.1740 0.03351 1.3369  0.006 **
Residual 37   4.8150 0.92754
Total    39   5.1912 1.00000
---
Signif. codes:  0 ‘***’ 0.001 ‘**’ 0.01 ‘*’ 0.05 ‘.’ 0.1 ‘ ’ 1
```

Supplementary Table S8. PERMANOVA unweighted UniFrac distances for generation 1. The R^2 values were computed using the PERMANOVA test with the adonis2 function for generation 1 for unweighted UniFrac distances. Genotype is referred to as “Lineage” and biomass treatment as “Regime”. Model (distance.matrix~biomass treatment +genotype).

```
adonis2(formula = unifrac ~ Regime + Lineage, data = meta, permutations = 999)
         Df SumOfSqs      R2      F Pr(>F)
Regime    1  0.20168 0.13113 2.5871  0.013 *
Lineage   1  0.40088 0.26065 5.1425  0.001 ***
Residual 12  0.93544 0.60822
Total    14  1.53799 1.00000
---
Signif. codes:  0 ‘***’ 0.001 ‘**’ 0.01 ‘*’ 0.05 ‘.’ 0.1 ‘ ’ 1
```

Supplementary Table S9. PERMANOVA unweighted UniFrac distances for generation 8. The R^2 values were computed using the PERMANOVA test with the adonis2 function for generation 8 for unweighted UniFrac distances. Genotype is referred to as “Lineage” and biomass treatment as “Regime”. Model (distance.matrix~biomass treatment +genotype).

Generation	Treatment	mean_BM	sd_bm
Gen1	High	33.755000	10.832288
Gen1	Low	33.755000	10.832288
Gen2	High	15.138000	5.784211
Gen2	Low	16.960000	5.073018
Gen3	High	5.758000	2.742120
Gen3	Low	5.406250	2.735613
Gen3	Control	6.385000	3.079862
Gen4	High	11.006383	4.991053
Gen4	Low	8.670833	3.810286
Gen4	Control	13.865000	6.194673
Gen5	High	16.673469	8.263140
Gen5	Low	12.648837	6.441989
Gen5	Control	17.485000	9.218761
Gen6	High	2.611111	1.740066
Gen6	Low	2.829167	1.734869
Gen6	Control	3.826667	1.445420
Gen7	High	16.888000	5.249865
Gen7	Low	12.110000	5.760713
Gen7	Control	21.700000	4.828733
Gen8	High	10.286000	6.848179
Gen8	Low	10.306250	6.745886
Gen8	Control	4.704545	5.052814

Supplementary Table S10. Mean and Standard deviation for biomass of L*er* for all generations. The raw value of mean biomass of L*er* are referred to as ‘mean_BM’ and the standard deviation is referred to as ‘sd_bm’.

Generation	Treatment	mean_BM	sd_bm
Gen1	High	20.730000	9.5565568
Gen1	Low	20.730000	9.5565568
Gen2	High	3.773469	2.0234846
Gen2	Low	3.906122	2.5696472
Gen3	Control	4.016667	1.8166712
Gen3	High	3.177778	1.1874129
Gen3	Low	3.085714	1.3985308
Gen4	Control	4.985714	3.5589232
Gen4	High	3.083333	1.6011360
Gen4	Low	2.313636	1.2305399
Gen5	Control	5.656522	4.0230954
Gen5	High	2.118919	1.3910104
Gen5	Low	2.035000	1.3098346
Gen6	Control	1.178571	0.8648509
Gen6	High	1.443590	0.9781279
Gen6	Low	1.619048	1.3096710
Gen7	Control	7.720000	4.4673258
Gen7	High	9.265217	4.7162234
Gen7	Low	7.244444	4.0908570
Gen8	Control	1.220000	1.8569988
Gen8	High	6.442105	3.3952774
Gen8	Low	2.145455	2.5654058

Supplementary Table S11. Mean and Standard deviation for biomass of Cvi for all generations. The raw value of mean biomass of Cvi are referred to as ‘mean_BM’ and the standard deviation is referred to as ‘sd_bm’.
